# A Randomized Crossover Clinical Trial Investigating 16‐Branched Filament Toothbrush Effects on Dental Plaque Removal

**DOI:** 10.1002/cre2.70192

**Published:** 2025-07-31

**Authors:** Nozomi Niki, Makoto Fukui, Ichika Sone, Kosuke Kataoka, Moeno Takeshita, Keisuke Kato, Daisuke Hinode

**Affiliations:** ^1^ Department of Hygiene and Oral Health Science Tokushima University Graduate School of Biomedical Sciences Tokushima Japan; ^2^ Planning Office, Sunstar Foundation Osaka Japan; ^3^ Department of Oral Health Science and Social Welfare Tokushima University Graduate School of Biomedical Sciences Tokushima Japan; ^4^ R&D, Sunstar Inc. Osaka Japan

**Keywords:** 16‐branched filament ends, dental plaque, gingival abrasion, toothbrush

## Abstract

**Objectives:**

Self‐care using an appropriate toothbrush is important for oral health management. The aim of this clinical trial in older adults was to evaluate the effectiveness of a prototype toothbrush with 16‐branched filament ends (16‐BFE) in dental plaque removal compared to a toothbrush with super tapered filament (STF).

**Material and Methods:**

Fifty older adults in Japan (13 males and 37 females, mean age 70.8 ± 8.7 years) with 20 or more teeth were enrolled in this study. Participants were divided into Group A (26 subjects) and Group B (24 subjects) and a randomized crossover clinical trial was conducted. Intervention Study I was performed for 3 min using a prototype 16‐BFE toothbrush. Dental plaque accumulation areas and gingival abrasion areas were measured before and after brushing by staining with MIRA‐2‐Ton. Intervention Study II was conducted in the same manner using an STF toothbrush. Crossover design was as follows: Group A was followed by an intervention study from I to II, while Group B was from II to I, with a washout period of 2 or 3 months. Analysis was performed using a modified PCR method (six sections of one tooth surface).

**Results:**

The average dental plaque removal rate using a 16‐BFE toothbrush was 22.4%, which was a significant reduction compared to 18.5% of the STF toothbrush (*p* < 0.05). There was only one small area out of 1700 areas examined regarding gingival abrasion when using a 16‐BFE toothbrush.

**Conclusion:**

These results suggested that the 16‐BFE toothbrush provided a more effective way in dental plaque removal when compared to the STF toothbrush and was less harmful to the gingiva. Even though 16‐BFE showed a significant improvement, clinical relevance and long‐term effects needed to be further investigated.

## 1 Introduction

The awareness of oral hygiene has increased in recent years; 79.2% of the population perform brushing their teeth two or more times a day in Japan, and this percentage is increasing year by year (Ministry of Health, Labour and Welfare: National Survey of Dental Diseases 2022). On the other hand, the percentage of people with periodontal pockets of 4 mm or more increases with age, partly due to the increase in the number of remaining teeth (Ministry of Health Labour and Welfare: National Survey of Dental Diseases 2022).

It is important to practice appropriate self‐care to maintain oral health throughout one's life. Brushing with a toothbrush is an effective and simple method of oral hygiene management (Deinzer et al. 2024; Axe et al. 2023). Currently, toothbrushes with a variety of handle shapes and bristles are available in the market (Otsuka et al. 2020). The bristles of the toothbrush are particularly important in determining the cleaning effect and feel of using it (Rahman 2023), therefore, the bristles' shape is selected according to the oral condition and age of the patient. Many elderly people have physical disabilities, such as reduced manual dexterity, that make brushing their teeth difficult (Razak et al. 2014). Therefore, there is a demand for toothbrushes that can effectively remove dental plaque without damaging the gums.

In recent years, super tapered filaments (STF: filament with thinned tips through chemical treatment), which are characterized by their soft feel when used, have been used in many toothbrushes currently in the market (Takeshita et al. 2022). STF are expected to be soft against the gingiva and easy to insert into the narrow space between teeth (Capopossi et al. 2016). In addition, it has been reported that they cause less abrasion to the gingiva when brushing (Versteeg et al. 2008). However, it has also been reported that STFs have low brushing efficiency (Takayanagi 2014), so they are recommended for patients who can take their time to brush carefully.

Branched bristles, which are currently under development and have 16‐branched filament ends (16‐BFE) with a very small diameter, so they are gentle on the gingiva like STF and can provide care for the gingiva. In addition, the diameter of the bristles excluding the tips is the same as the diameter at the base of the bristles, so the elasticity of the entire bristles is maintained, and it is expected that they will be able to efficiently remove dental plaque that has accumulated on the tooth surface. Therefore, it is presumed that branched bristles will contribute to oral hygiene in people whose dental diseases increase with age. Clinical studies have been conducted on STF and rounded bristles with rounded tips (Capopossi et al. 2016; Versteeg et al. 2008; Ren et al. 2007; Hoogteijling et al. 2018) but no studies have been conducted on toothbrushes using branched bristles.

The aim of this crossover clinical trial was to evaluate the effectiveness of a prototype 16‐BFE toothbrush in dental plaque removal compared to an STF toothbrush. The novelty of this study is the investigation of the 16‐BFE toothbrush.

## 2 Materials and Methods

### 2.1 Subjects and Target Teeth

The study was conducted in Tokushima University Hospital with an elderly population of 50 years and older to investigate the effectiveness of toothbrushes with branched filament ends. Participants had been visiting the dental section for more than 1 year and receiving continuous brushing instructions from dental hygienists. Inclusion criteria were those who had 20 or more teeth and provided consent to participate in the study. Exclusion criteria were those who had all molars missing, half or more of their teeth were full or 3/4 crowns, and those who were allergic to the components of the plaque staining solution. Target teeth were all teeth other than the excluded teeth. The excluded teeth were third molars, missing teeth, teeth with extensive crowns that would interfere with the evaluation, implant teeth, teeth with a large amount of dental calculus, and teeth that could not be measured due to severe malalignment. Additionally, subjects with movement restriction in the hands or arms were excluded. Regarding the study period, recruitment to participate in the study began on February 16, 2023, and the study ended on December 5, 2023.

### 2.2 Test Toothbrush

The toothbrush with 16‐branched filament ends (16‐BFE) used in the test was developed by Sunstar Inc. (Osaka, Japan). Super tapered filaments (STF) were used for the bristles in the control toothbrushes. The handles are the same as those of the Gum Plus Dental Brush (#366, Sunstar Inc.). The specifications of the 16‐BFE and STE are shown in Table 1. All test toothbrushes used in this study were provided by Sunstar Inc. The branched bristles, which have 16 filaments at the tip, have a very small diameter targeting of 0.018 mm, making them gentle on the skin, and the diameter of the bristles excluding the tip (approximately 0.3 mm from the tip) is the same as the diameter at the base of the bristles, maintaining the elasticity of the entire bristles. Although the deflection resistance value is slightly higher for 16‐BFE than that of STF, all values are smaller than those of the “Soft type toothbrush” in the previous study (Kaneyasu et al. 2020). The toothbrushes used in this study were subjected to material tests and elution tests based on the Food Sanitation Act as a safety evaluation, and it was confirmed that there were no problems.

**Table 1:** Test toothbrushes specification.

Toothbrush		16‐BFE	STF
Appearance (handle)		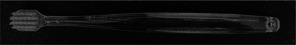	
Handle	Material	Saturated polyester resin	
	Length	180.0 mm	
	Number of holes	22 holes	
	Hole diameter	1.5 mm	
	Head thickness	4.0 mm	
	Head width	11.3 mm	
Appearance (form of filament implant)		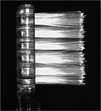	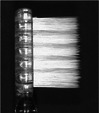
Filament tip shape		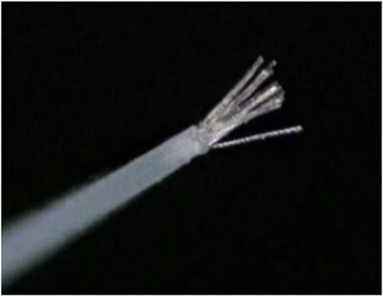	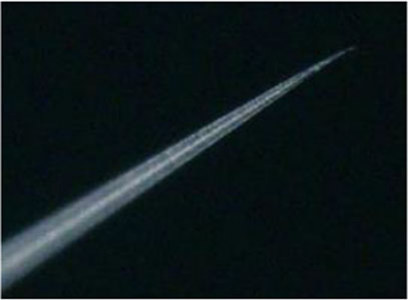
Filament	Material	Saturated polyester, nylon	Saturated polyester
	Length	10.5 mm	10.5 mm
	Diameter	0.170 mm	0.178 mm
Resistance of tufted portion to deflection^a^		2.92 ± 0.06 N/cm²	2.67 ± 0.07 N/cm²

^a^ Data were obtained by measuring the resistance of the tufted portion to deflection according to the method of the International Organization for Standardization (ISO) 22254.

### 2.3 Study Design

This study was designed as a randomized crossover comparative study. This study design was listed according to the CONSORT 2010 statement protocol. The sample size was determined as follows. As a preliminary clinical trial, the dental plaque removal rate by brushing for 3 min was examined for 8 employees of Sunstar company using the test and control toothbrushes mentioned above. The resulting effect size of 0.537 was entered into G*Power (G*Power 3.1 Manual 2023) with a detection power level of 0.8, and it was determined that 44 or more subjects were necessary.

**Figure 1:**
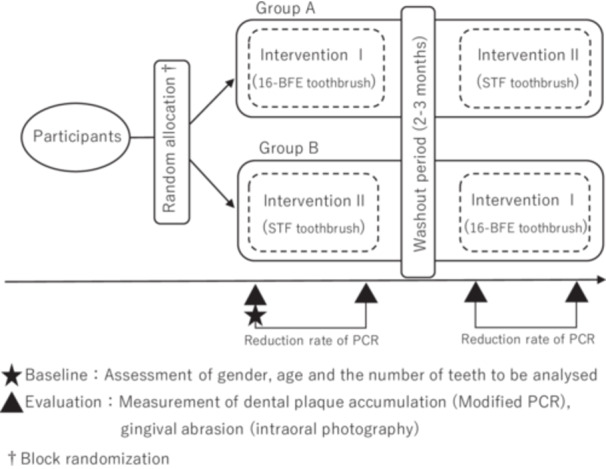
Outline of the crossover clinical trial.

Figure 1 shows the outline of the crossover clinical trial in this study. Subjects were randomly assigned to Group A and Group B. Group allocation was performed by someone other than the examiner using block randomization (Lim and In 2019). The crossover clinical trial was conducted in the order from intervention I to intervention II for Group A and from intervention II to intervention I for Group B, with a washout period of 2 to 3 months. The washout period was determined according to a preliminary study, and no significant carryover effect was observed during the 3‐week washout period. The intervals between intervention test I and intervention test II were set to match the intervals between the dental visits of the subjects. The closed triangle in Figure 1 shows the time of evaluation in this intervention study.

For the intervention Study I, the protocol of the clinical trial is as follows: (a) oral hygiene was stopped after dinner the day before the clinical trial. (b) Dental plaque attached to the tooth surfaces was stained with plaque staining solution (Mira‐2‐Ton, Hager & Werken, Germany) in the morning on the day of the study and the interdental papillary, marginal and attached gingiva between the labial surfaces of the first premolar were also stained. (c) After rinsing once with water, an angle wider was attached and intraoral photographs were taken using a digital camera (Eye Special C‐II, Shofu, Kyoto, Japan), (d) accumulation of dental plaque was measured using a modified Plaque Control Record (PCR) method (Ogawa et al. 2006). Then, (e) tooth brushing was performed by 16‐BFE toothbrush for 3 min without using a hand mirror and toothpaste. The method of tooth brushing was not specified, and each participant was allowed to brush their teeth in any way. (f) After brushing for 3 min, the dental plaque and gingiva were stained with Mira‐2‐Ton again, (g) intraoral photographs were taken, and the accumulation of dental plaque was measured again using a modified PCR method. Intervention study II followed the same procedure as intervention study I, with an STF toothbrush used in step (e). Examiners and subjects were blinded to which toothbrush was used. Clinical trials and analyses of plaque removal rates were all carried out by the researchers.

### 2.4 Evaluation of Items

**Figure 2:**
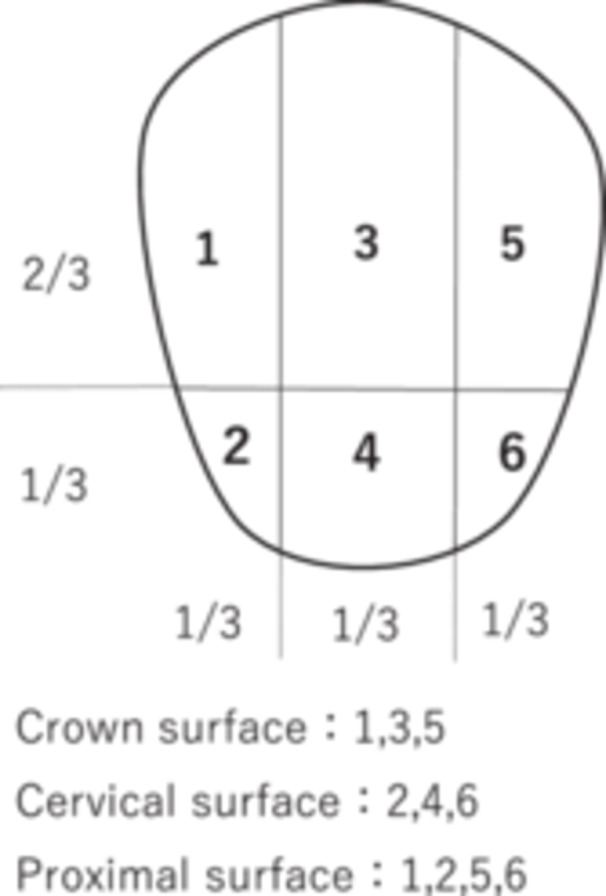
Modified plaque control record (PCR).

1. **Evaluation of dental plaque removal**
  The primary evaluation item was the reduction rate of dental plaque. After staining, the accumulation of dental plaque on the tooth surface was measured using a modified PCR method. As shown in Figure 2, each buccal and lingual surface was divided into 6 sections. Before the study began, examiners underwent thorough training in judging the results using photographs. The rate of change in the measurement value before and after brushing was calculated as the reduction rate of PCR using the following formula:
  Reduction rate of PCR (%) = [(dental plaque accumulation site before brushing − dental plaque accumulation site after brushing)/dental plaque accumulation site before brushing] × 100
2. **Evaluation of gingival abrasions**
  As a secondary evaluation item, the Gingival Abrasion Score (GAS) (Danser et al. 1998) was used to evaluate gingival abrasions on the labial surfaces of the upper and lower anterior teeth using intraoral photographs taken during the study. GAS measurements were performed on photographs taken before and after brushing, and the increase in the number of abrasions on the interdental papillary, marginal, and attached gingiva was evaluated. The size of gingival abrasions was measured according to size, with small: ≤ 5 mm and large: > 5 mm.
3. **Other evaluation items**

Other evaluation items of subjects such as gender, age, the number of remaining teeth, and the number of teeth for measurement of dental plaque accumulation at the baseline were also analyzed.

### 2.5 Statistical Analysis

Comparisons of baseline items were made using the Mann–Whitney *U* test or Student's *t*‐test, and comparisons between genders were made using the chi‐square test. The Wilcoxon signed‐rank test was used to compare the PCR ratio between before and after brushing in both Intervention I and II. The Mann–Whitney *U* test was used to analyze the carryover effect and period effect of the crossover study, and to compare the reduction rate of PCR between two groups. The kappa coefficient was used to confirm the degree of agreement between the dental plaque accumulation sites by the two evaluators. Statistical analysis was performed using IBM SPSS Statistics version 28 (IBM Japan Inc., Tokyo), and the statistical significance level was set at 0.05.

### 2.6 Ethics Approval

The method and objectives of this study were explained to the patients in Tokushima university hospital, who provided written informed consent before their participation in the study. The Ethics Committee of Tokushima University Hospital approved this study (Protocol approval number 4302).

## 3 Results

### 3.1 Characteristics of Subjects

The number of subjects who met the inclusion criteria and gave written consent was initially 53, but three of them dropped out during the clinical trial due to personal reasons or difficulty in visiting the university hospital; therefore, they were excluded from the analysis. The final number of subjects was 50 (13 male, 37 female, mean age 70.8 ± 8.7 years). Comparison of each item at baseline for each subject group is shown in Table 2. The mean and standard deviation of all subjects at baseline were 25.7 ± 2.2 for the number of current teeth, 21.6 ± 4.1 for the number of test teeth, 76.5 ± 14.2% for modified PCR before brushing, and 32.4 ± 16.3% for the average of last three PCR, respectively. There were no differences in the values in each item at baseline between Group A and Group B. When the agreement of plaque accumulation sites by the two evaluators was confirmed, the kappa coefficient was 0.63, which was evaluated as having good reliability.

**Table 2:** Comparison of items at baseline.

Gender ratio	All subjects (50)					Group A (26)					Group B (24)					*p* value
	Males: females (13:37)					Males: females (6:20)					Males: females (7:17)					
	Mean value	Standard division	Median value	Percentile		Mean value	Standard division	Median value	Percentile		Mean value	Standard division	Median value	Percentile		
				25	75				25	75				25	75	
Age	70.8	8.7	73.0	66.5	76.0	69.4	9.9	72.0	60.5	76.3	72.4	7.1	74.0	67.3	76.0	0.236^a^
The number of present teeth	25.7	2.2	26.0	24.0	27.3	25.5	2.2	26.0	23.8	27.0	25.9	2.2	26.5	24.3	28.0	0.527^b^
The number of subject teeth	21.6	4.1	21.5	19.8	24.0	21.6	4.4	21.0	19.0	24.5	21.6	3.9	23.0	20.0	24.0	0.996^a^
Modified PCR before brushing (％)	76.5	14.2	80.3	70.6	86.8	76.4	16.2	82.1	70.1	87.3	76.6	12.0	79.9	70.8	86.1	0.484^b^
Average of the last three O'Leary's PCR (％)	32.4	16.3	28.5	19.9	45.1	31.9	13.6	27.6	22.9	45.1	32.9	19.2	29.8	18.8	45.6	0.832^a^

^a^ Student's *t*‐test.

^b^ Mann–Whitney *U* test.

### 3.2 Carryover Effect and Period Effect

In a crossover study, it is necessary to consider “the carryover effect” which is defined as the lingering effect of the treatment of the previous study period on the current study period, and “the period effect” which represents a systematic difference between different periods in the outcome for evaluating treatment. Therefore, the influence of the above on intervention studies I and II was examined. As a result of the analysis, no carry‐over effect was observed, but the period effect showed differences in the reduction rate of PCR on the whole tooth surface, cervical surface, and proximal surface (Table 3).

**Table 3:** Carryover effect and period effect on plaque removal rate in a crossover trial.

	Whole tooth surface	Crown surface	Cervical surface	Proximal surface
Carryover effect	0.522	0.786	0.534	0.741
Period effect	0.026*	0.322	0.003**	0.014*

The value means the *p*‐value by statistical analysis.

Mann‐Whitney *U* test.

* *p* < 0.05

** *p* < 0.01.

### 3.3 Comparison of the Reduction Rates of PCR

**Figure 3:**
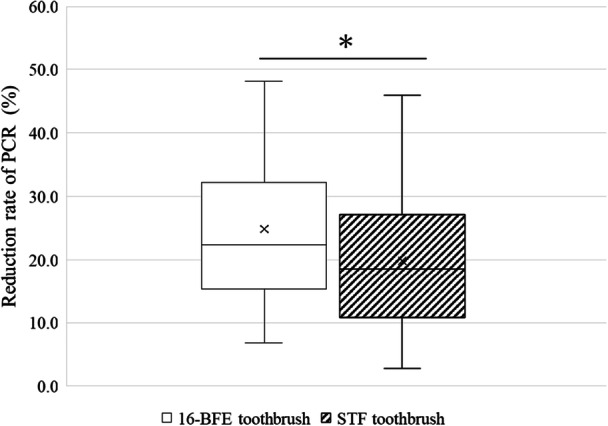
Reduction rate of PCR (whole tooth surface). Mann–Whitney *U* test, *p < 0.05.

**Figure 4:**
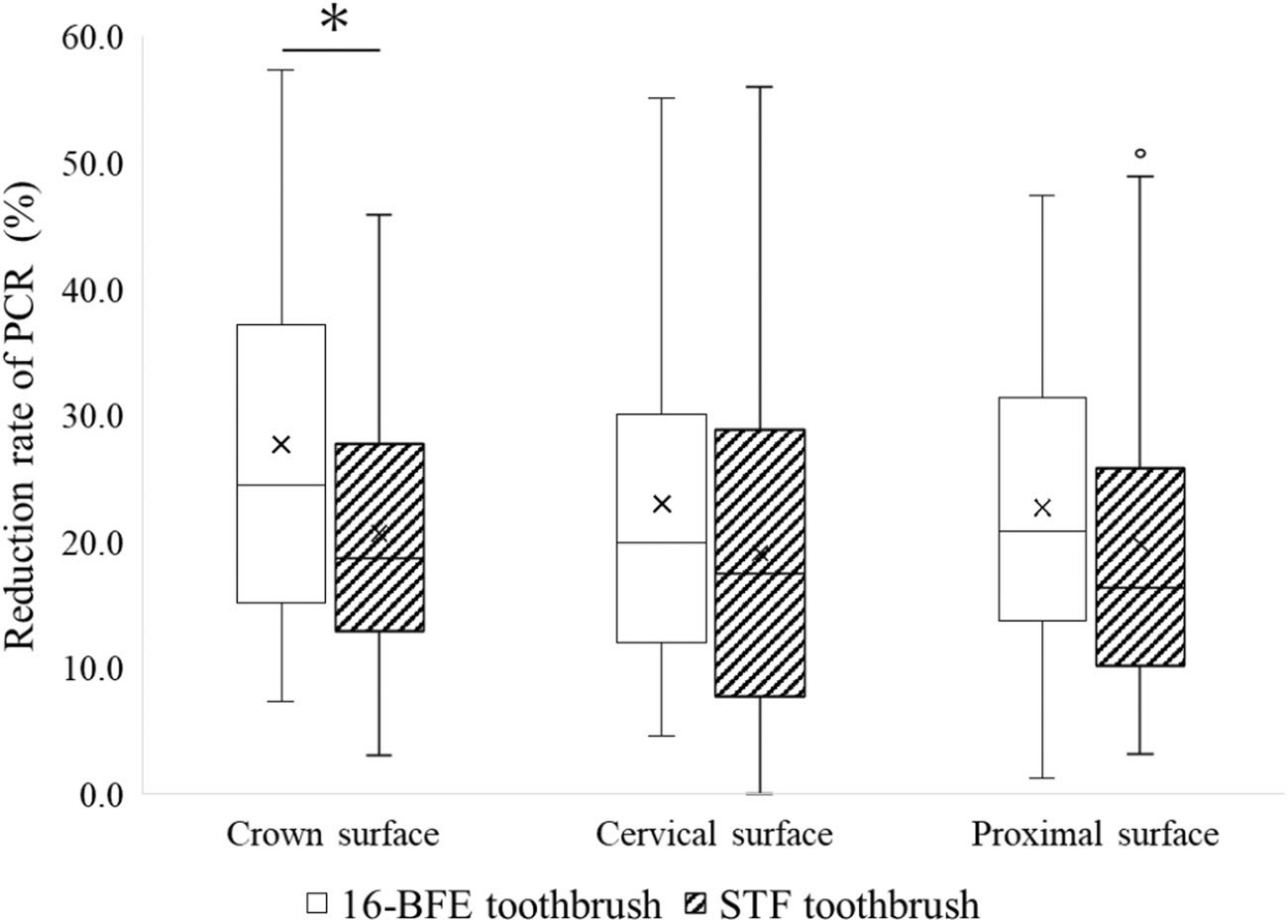
Site‐specific reduction rate of PCR. Mann–Whitney *U* test, *p < 0.05.

The PCR ratio between before and after brushing was significantly reduced (*p* < 0.01) on the whole tooth surface, the crown, cervical, and proximal surfaces in both Intervention I and II by the Wilcoxon signed‐rank test, respectively. Figures 3 and 4 show the results of the reduction rate of PCR. Correlation coefficient calculated from Z score and number of subjects was used for effect size. The effect sizes were estimated as follows: absolute value > 0.1 is a small effect, > 0.3 is a medium, and > 0.5 is a large, respectively (Brydges 2019). The median (95% confidence interval) plaque adhesion site reduction rate was 22.4% (17.3–29.2) for the 16‐BFE toothbrush and 18.5% (14.2–23.5) for the STF toothbrush, and the 16‐BFE toothbrush was significantly higher than the STF toothbrush on the whole tooth surface in Figure 3 (*p* = 0.028, effect size = −0.22).

For the site‐specific reduction rate of PCR, each site divided by the modified PCR method was analyzed. The analysis results of the crown, cervical, and proximal surfaces are shown in Figure 4. In the crown, the median of reduction rates of PCR for the 16‐BFE toothbrush and the STF toothbrush were 24.5% (20.8–33.8) and 18.7% (16.7–22.4), respectively, and a significant difference was observed (*p* = 0.016, effect size = −0.24). On the other hand, in the cervical region, the median of reduction rates of PCR were 19.9% (16.9–26.6) and 17.5% (12.3–22.5), (*p* = 0.126, effect size = −0.15) and in the proximal surface, the median of reduction rates of PCR were 20.8% (15.0–26.7) and 16.3% (11.8–21.8) (*p* = 0.065, effect size = −0.18), respectively. The 16‐BFE toothbrush had a higher reduction rate than those of the STF toothbrush; however, no significant difference was observed.

### 3.4 Evaluation of Gingival Abrasions

Among the areas that could be determined from intraoral photographs, increased gingival abrasions were only observed in one small (≤ 5 mm) area out of 1700 areas examined when using a 16‐BFE toothbrush. None were observed when using an STF toothbrush.

## 4 Discussion

In previous related studies, the clinical effectiveness of STF and rounded bristles has been compared; however, toothbrushes with branched filament ends have not been examined (Razak et al. 2014). While the use of STF toothbrushes is increasing among elderly people and patients with periodontal disease, STF possesses problems with the dental plaque removal rate. In this study, we considered that a 16‐BFE toothbrush, which shares a common feature of STF in terms of its gentle feel, could solve the problem of low dental plaque removal rates. When comparing 16‐BFE and STF toothbrushes, it was found that the reduction rate of PCR on the whole tooth surface by the 16‐BFE toothbrush was significantly higher than that with an STF toothbrush.

In the case of a 16‐BFE toothbrush, the diameter of the bristles, excluding the tip (branched part 0.3 mm from the tip), is the same as the diameter at the base of the bristles, so the elasticity of the bristles is maintained. A preliminary in vitro study confirmed that the 16‐BFE toothbrush has higher elasticity than the STF toothbrush, whose bristles have a smaller diameter toward the tip, suggesting that effective plaque removal is possible. Furthermore, a preliminary in vitro study showed that the 16‐BFE toothbrush efficiently removed high‐viscosity pseudo‐plaque (data not shown). The durability of the two filaments was compared in a preliminary study (Supplemental Figure 1). After 10,000 repeated strokes, damage was observed at the tips of both filaments. The effect of damage seemed to be greater for STF than for 16‐BFE. Furthermore, the difference in cost per toothbrush between the two was only 3 Japanese yen, which means there is almost no cost difference. Therefore, in this study, it seemed that the subdivided bristle shape of the 16‐BFE toothbrush enabled efficient plaque removal.

It revealed that the 16‐BFE toothbrush showed a higher reduction rate of PCR than the STF toothbrush in all sites, including the crown, cervical, and proximal surfaces; however, a significant difference was only observed in the crown surface. The shape of the crown surface is less uneven and less complex than the other two sites, and the environment that makes plaque removal easier, which is thought to have made a significant difference more likely to occur.

Rosema et al. (2014) reported that an average of 15 or more gingival abrasions were observed per person when using a manual toothbrush. Moreover, Capopossi et al. (2016) reported that an average of 6.7 gingival abrasions were observed per person when using a tapered bristle toothbrush. Gingival abrasions in these studies were evaluated in the whole alignment of the mouth. Only one area was observed as gingival abrasions by a 16‐BFE toothbrush, and none were observed with an STF toothbrush, whereas the labial surfaces of the upper and lower anterior teeth were observed in this study. Even considering that only the labial surfaces of the anterior teeth were evaluated in this study, the number of gingival abrasions in this study was small. These results suggest that both the 16‐BFE toothbrush and STF toothbrush used in this study were gentle on the gingival tissue.

Although not shown in the results, the relationship between the average O'Leary's PCR (O'Leary et al. 1972) values from the last three regular maintenance sessions of the subjects and the removal rate for the entire tooth surface using a branched bristle toothbrush was examined. The rank correlation coefficient was −0.434 (*p* = 0.001), indicating a statistically significant negative correlation. Therefore, there was a tendency for people with normally high PCR values to have a lower removal rate in this study. It has been reported by Tsubosaki et al. (2021) that the difference in proficiency in each subject affects the removal rates of dental plaque. The results of this study might support their results.

Regarding the study design, there was no carryover effect from the crossover study, so it is believed that a sufficient washout period was set. However, the period effect was observed on the reduction rate of PCR in the whole tooth surface, cervical surface, and proximal surface. Normal brushing was instructed in each intervention process in this study. However, the period effect is thought to be due to the subjects becoming accustomed to brushing without looking in a mirror and being able to brush more effectively for 3 min based on their experience in the first intervention study. This is thought to be due to the subjects becoming accustomed to the procedure of the test method and were able to concentrate more on brushing during the second intervention than during the first intervention. Many people brush using a mirror at home or brush for longer than 3 min, so it is thought that the reduction rate of PCR was higher in the second intervention study when participants were accustomed to this procedure. Since no period effect was observed in the reduction rates of PCR in the crown surface, it is thought that the period effect does not affect the effect due to differences in bristle tip shape in the crown surface.

## 5 Limitation

A limitation of this study is that the reduction rate of PCR in this study was lower than that in previous studies (Takeshita et al. 2022), those removal rate of dental plaque were 40‐60%. The subjects of this study were elderly people with an average age of 70.8 years old. It has been reported that plaque accumulation in elderly people is worsened by dental restorations, removable dentures, and gingival recession, and is also associated with decreased eyesight and decreased manual dexterity (Razak et al. 2014). Oral hygiene management becomes difficult due to a decline in the oral environment and physical function. Therefore, it is thought that the brushing time (3 min) for all tooth surfaces in older adults of this study might be short. Other studies comparing toothbrush removal rates have set brushing time to 5 min (Muraoka et al. 2011), and it is possible that the short brushing time affected the low reduction rate of PCR. The effect size of 5 min brushing tended to be smaller than that of 3 min in the preliminary study. It is necessary to set the brushing time according to the characteristics of the subjects and the toothbrush in future studies.

In addition, the second intervention study was timed to coincide with the interval between patients' visits (2–3 months), the interval between the dental plaque‐free state achieved by PMTC and the test date was longer than in previous studies (Takeshita et al. 2022) (1–4 weeks). Therefore, removal of old plaque due to roughening of the tooth surface may be difficult with short brushing time. Another limitation of this study is that we were unable to measure the subjects' motor function or manual dexterity levels. Although the subjects of this study were elderly, we did not evaluate non‐carious cervical lesions (NCLL) in areas of exposed roots due to gingival recession. Because NCLL is thought to affect plaque control in the cervical area, future studies should also evaluate the impact of NCLL.

For elderly people, the brushing area increases due to gingival recession, and manual dexterity declines with age (Razak et al. 2014). There is a demand for the development of toothbrushes that can effectively remove plaque without damaging the gums. In this study, a company and a research institute jointly conducted clinical trials to verify the effectiveness of a toothbrush developed to satisfy these needs. A part of this study was funded, and the newly developed toothbrushes were provided by the company. However, the clinical trials and analysis of plaque removal rates were all carried out by the researchers of university members; therefore, conflicts of interest that constitute research misconduct are not permitted. Clinical relevance still needs further studies. This study was conducted on elderly Japanese people who regularly received brushing instructions, so the results cannot be generalized. Future studies should consider the level of manual dexterity in older adults, evaluate microtrauma after long‐term use, and include a wider range of age groups worldwide to establish the effectiveness of the 16‐BFE.

## 6 Conclusions

These results suggested that the toothbrush with 16‐branched filament ends provided a more effective way in dental plaque removal when compared to the toothbrush with super tapered filament and was less harmful to the gingiva. Even though 16‐BFE showed a significant improvement, clinical relevance and long‐term effects needed to be further investigated.

## Author Contributions

Nozomi Niki, Makoto Fukui, and Daisuke Hinode designed, coordinated, and performed the clinical trial and drafted the paper. Ichika Sone and Kosuke Kataoka performed the clinical trial and drafted the paper. Moeno Takeshita and Keisuke Kato designed, performed the statistical analysis, and drafted the paper. All authors reviewed the paper critically for content and approved it for submission.

## Conflicts of Interest

Nozomi Niki, Makoto Fukui, Ichika Sone, and Kosuke Kataoka report no conflict of interests. Daisuke Hinode received financial support by KAKENHI Grant Number 23K09481 from the Japan Society for the Promotion of Science. Moeno Takeshita and Keisuke Kato are employees of Sunstar Inc. and part of this study was funded by Sunstar Inc. The company provided the newly developed toothbrushes.

## 1 Data Availability Statement

The authors confirm that the data supporting the findings of this study are available within the article. The data that support the findings of this study are available from the corresponding author upon reasonable request.

## Supporting information


**Supplemental Figure 1:** Durability of the filament.
