# Supplementary material for: A Randomized Crossover Clinical Trial Investigating 16‐Branched Filament Toothbrush Effects on Dental Plaque Removal
Source: Clin Exp Dent Res. 2025 Jul 31;11(4):e70192. doi: 10.1002/cre2.70192 (PMC12311836; doi:10.1002/cre2.70192)
Supplement: Supplementary file 1 — Supplemental Figure 1: Durability of the filament. [file CRE2-11-e70192-s001.docx]

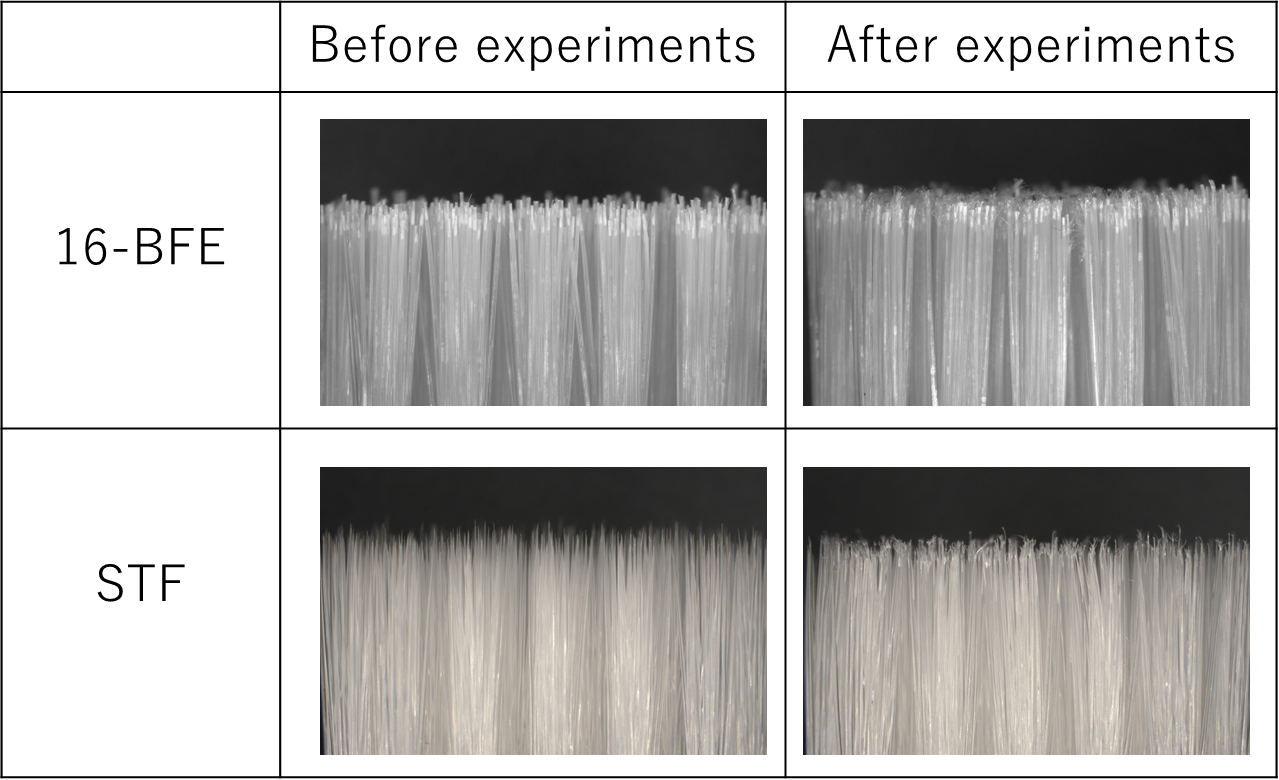


**SUPPLEMENTAL FIGURE 1** Durability of the filament

Experiments for the durability of filament were performed according to the method of ISO 11609 as follows: Testing equipment; toothbrush durability testing machine set to apply a load of 200 g to the head, Number of strokes; 10,000 round trips immersed in water at 37±3 °C, Brushing subject; artificial dentition model.
